# Are Individual Differences in Information-Processing Styles Related to Transformational Leadership? A Test of the Cognitive Experiential Leadership Model

**DOI:** 10.3389/fpsyg.2021.599008

**Published:** 2021-03-26

**Authors:** Guy J. Curtis, Serena Wee

**Affiliations:** School of Psychological Science, University of Western Australia, Perth, WA, Australia

**Keywords:** transformational leadership, thinking styles, Cognitive Experiential Self-Theory, behavioral coping, rational, Cognitive Experiential Theory

## Abstract

The recently proposed Cognitive Experiential Leadership Model (CELM) states that leaders’ preference for rational thinking and behavioral coping will be related to their level of transformational leadership. The CELM was based on research that principally used cross-sectional self-report methods. Study 1 compared both self-ratings and follower-ratings of leadership styles with leaders’ self-rated thinking styles in 160 leader-follower dyads. Study 2 compared both self-ratings and coworker-ratings of leadership styles with leaders’ self-rated thinking styles for 74 leaders rated by 607 coworkers. In both Studies, leaders’ rational thinking, imaginative thinking, and behavioral coping correlated positively with their self-rated transformational leadership. However, only behavioral coping, but not rational thinking, was correlated with follower-rated (FR) transformational leadership in Study 1, and thinking styles were unrelated to other-rated transformational leadership in Study 2. These results partly support and partly challenge the CELM. Practically, this study suggests that leadership may be improved by leaders developing their capacity for behavioral coping.

## Introduction

Would anyone argue with the proposition that leaders who think better are likely to lead better? In order to be testable, however, this proposition requires a definition of good thinking and good leadership. Recently, Cerni et al. (2014) proposed that individual differences in thinking styles are related to leaders’ tendency to employ more, or less, effective leadership styles and behaviors. Their proposed model, the Cognitive Experiential Leadership Model (CELM), predicts that there should be connections between individual differences in the ways in which leaders tend to think and the ways in which they will tend to lead. This model makes a potentially interesting contribution to the leadership literature because, as semi-malleable traits, thinking styles, if connected with leadership, may be assessed for selection of leaders and developed in order to enhance leadership (Cerni et al., 2014). Concretely, if leadership style is related to thinking style, then the potential exists to develop leaders by developing how they think. Despite this potential, to date, several of the possible connections between individual differences in thinking styles and leadership have not been adequately tested empirically.

To begin, it is important to explain the CELM and the two theories that Cerni et al. (2014) integrated in this model: Cognitive Experiential Theory (CET; Epstein, 2014; previously known as Cognitive-Experiential-Self Theory or CEST, Epstein, 1994) and Full-Range Leadership Theory (Bass, 1997). CET provides a framework for understanding individual differences in thinking styles and the consequences of these for personality and behavior. The Full-Range Leadership Theory proposes the existence of three main styles of leadership, the most effective and most researched of which is *transformational leadership* (Judge and Piccolo, 2004; Dinh et al., 2014).

## Full-Range Leadership Theory

Leadership theories are legion, as are the styles of leadership described within them. Still, many effective leadership behaviors and methods are captured by the term transformational leadership (Bass and Avolio, 1997). Transformational leaders identify required changes and motivate and inspire their followers to work toward superordinate goals for the good of the group or the organization (Bass, 1997; Curtis, 2013). In addition, transformational leaders extend followers beyond their own self-interest through intellectual stimulation and individual consideration (Bass and Avolio, 1997). Despite some well-argued criticism (e.g., van Knippenberg and Sitkin, 2013), transformational leadership has attracted, and continues to attract, substantial attention among researchers and practitioners because of the positive practical outcomes for organizations when leaders use this style (Judge and Piccolo, 2004; Dinh et al., 2014). Within the full range leadership theory, transformational leadership has been contrasted with transactional leadership, and passive-avoidant (Avolio et al., 1999) and/or laissez-faire leadership styles (Bass and Avolio, 1994).

Transactional leadership involves exchanges such as rewards and punishments between leaders and followers (Bass and Avolio, 1994). In contrast, passive-avoidant and laissez-faire leadership are non-interventionist approaches to leadership characterized by inaction, or excessively delayed action, from leaders (Bass and Avolio, 1994; Avolio et al., 1999). Research demonstrates that transformational leadership produces more effective outcomes for organizations than transactional leadership; and both transformational and transactional leadership out-perform passive-avoidant or laissez-faire leadership (Judge and Piccolo, 2004). Although these leadership style descriptions suggest how more, and less, effective leaders may be expected to behave, the full-range leadership theory is silent on how leaders think.

## Cognitive Experiential Theory

Numerous theories in psychology are based on the idea that people process information using two systems: one system that is conscious, logical, slow, and rational, and another system that is fast, intuitive, non-conscious, and affect-laden, but potentially more prone to systematic bias (Gilovich et al., 2002; Wilson, 2002). Epstein (1994) developed a theoretical and empirical framework for assessing individual differences in information-processing styles that attempts to capture people’s preference for, and skill in, using these different systems. CET was developed as a theory of personality, integrating psychological theories of cognitive processes with measurable individual differences in behavioral tendencies (Epstein, 1994, 2014).

Epstein (1994) called the dual information-processing systems the *rational system* and the *experiential system*. CET proposes that the extent to which people tend to think consciously and rationally vs. non-consciously (relying on experience), and underlies most behavior. Like other dual-process theories, according to CET rational thinking is slow, conscious, relatively affect-free, and more useful in novel situations. In contrast, the experiential system is defined as a broad intuition-based system that encapsulates emotion, concrete reasoning, generalization, spontaneity, and imagination (Norris and Epstein, 2011). Moreover, how effectively the experiential system is used is described in the sub-theory of *constructive thinking* (Epstein, 1998, 2001).

Constructive thinking is defined as automatic thinking that occurs with a minimum cost in stress, which is contrasted with people’s potential to think destructively, i.e., in ways that create or exacerbate stress (Epstein, 1998). An important component of constructive thinking to define for the purposes of this paper is *behavioral coping*, which is a preference for realistic optimism, conscientiousness, and taking action in the face of problems. Behavioral coping has been linked with effective leadership and is a key variable in the CELM (Cerni et al., 2014).

## Cognitive-Experiential Leadership Model

Cerni et al. (2014) proposed the CELM as a way to capture the alignment of individual differences in thinking styles, as described by CET with leadership styles, as outlined in the Full-Range Leadership Theory, and leadership behaviors (specifically influence and conflict management). An important conceptual underpinning for the CELM is that leadership is not a one-shot behavior, but a series of interactions and behaviors over time. Because of this, Cerni et al. (2014) argue that understanding how people *tend* to think will provide a good guide to how they *tend* to lead and make decisions; this will determine what their behavior as a leader will *tend* to be like over time. The CELM predicts that the rational thinking, behavioral coping, imagination, and emotionality elements of leaders’ thinking styles will predict transformational leadership.

### Rational Thinking and Transformational Leadership

There are two main theoretical reasons why leaders’ preference for rational thinking should be related to their use of a transformational leadership style. First, rational thinking may be exhibited in leadership behavior akin to the transformational leadership factor of intellectual stimulation. Intellectual stimulation, as a factor of transformational leadership, is associated with the use of rational persuasion as an influence tactic (Charbonneau, 2004), and people who prefer rational thinking prefer rational persuasion as an influence tactic in the workplace (Curtis and Lee, 2013). Second, rational thinking is associated with several other psychological variables that are related to good leadership *per se* and transformational leadership specifically. Rational thinking is positively associated with intelligence, adaptability, and conscious self-awareness, all of which are associated with transformational and/or effective leadership (Epstein, 1998; Van Vugt, 2006; Yukl and Mahsud, 2010; Steele and Day, 2018).

Higher level leaders show stronger preferences for rational thinking (Akinci and Sadler-Smith, 2013), and several studies have found significant positive correlations between leaders’ self-rated rational thinking and their self-rated transformational leadership (e.g., Cerni et al., 2008; Curtis et al., 2017). In addition, leaders who are perceived as more rational by their followers are also rated as more transformational (Curtis, 2020). However, a methodological limitation of this existing evidence is the use of purely self-rated or purely other-rated cross-sectional methods, which may have inflated correlations due to common methods variance. Nonetheless, based on the theoretical connection between rational thinking and transformational leadership and the evidence from self-report studies, it is predicted that:

*H1*: Transformational leadership will be positively correlated with leaders’ preference for rational thinking.

### Behavioral Coping and Transformational Leadership

The CELM proposes that behavioral coping will be related to transformational leadership because behavioral coping is associated with cognitive and behavioral adaptability (Cerni et al., 2014). Behavioral coping is related to proactive action-focus problem solving, which may be exhibited in leaders’ dynamic, charismatic, and motivational overt behaviors that may appear as being transformational to followers. Moreover, behavioral coping is associated with low stress, allowing leaders to remain calm and employ their intelligence in decision making and interactions with followers (Fiedler and Garcia, 1987).

Several studies have found positive relationships between leaders’ tendency to use behavioral coping and both self-rated (Humphreys and Zettel, 2002; Cerni et al., 2008; Reynolds and O’Dwyer, 2008; Curtis et al., 2017) and other-rated transformational leadership (Atwater and Yammarino, 1993; Dubinsky et al., 1995; Cerni et al., 2010a,b). It is predicted that:

*H2*: Transformational leadership will be positively correlated with leaders’ preference for behavioral coping.

### Imagination, Emotionality, and Transformational Leadership

In addition to articulating connections between thinking styles and leadership styles that have been observed in the empirical literature, the CELM proposes further connections between thinking styles and leadership that have not yet been well-tested. For example, it proposes that the emotional and imaginative components of experiential thinking will be related to transformational leadership, because transformational leaders tap into followers’ emotions to motivate them toward an imagined ideal or goal (Cerni et al., 2014; Curtis and Cerni, 2015). Thus far in the empirical literature, the only studies to examine the possible relationships between imagination, emotionality, and transformational leadership are two studies by Curtis et al. (2017). Curtis et al. (2017) found that imagination predicted variance in leaders’ transformational leadership beyond that predicted by rational thinking and behavioral coping, but they found no relationship between emotionality and transformational leadership. Similarly, Curtis (2020) found that followers who evaluated their leaders as more imaginative also evaluated their leaders as more transformational. However, these previous studies only used other-report *or* self-report methods and these relationships are yet to be tested where thinking styles are leader-self-rated and leadership style is evaluated by others. Thus, as a test of the theory’s predictions it is expected that:

*H3*: Imagination will be positively correlated with transformational leadership.
*H4*: Emotionality will be positively correlated with transformational leadership.

## Study 1

Because previous research on connections between thinking-styles and leadership styles that test CELM used mostly cross-sectional self-reports, in the present study, leader-follower dyads were recruited so that leadership ratings could be obtained from followers. Given that thinking style is preferably self-rated, leaders rated their own thinking styles, and, to replicate past research leaders’ self-ratings of their leadership styles were also collected.

### Method

#### Participants and Procedure

A student-recruited samples methodology was used, with a total of 34 graduate and honors students at Murdoch University in Perth Australia tasked with recruiting a convenience sample of pairs of leaders and followers from among their personal contacts. A recent meta-analysis found that student-recruited samples in organizational research produced data with comparable representativeness to samples recruited in other ways (Wheeler et al., 2014). Additionally, a research assistant recruited further participants *via* email contact with alumni of Murdoch University Executive Education. Research participants were required to be working, over 18 years of age, and leaders and followers were required to have worked together for a minimum of 6 months.

Each participant was issued with a unique code number that they entered when completing measures online. Leader and follower code numbers were retained by the researcher so that paired data could be matched. A total of 245 leaders and 239 followers, among whom there were 191 matched pairs completed online questionnaires. Thirty-one pairs were omitted from the sample because of incomplete responses (>5%) or for falling outside the acceptable bounds of the Constructive Thinking Inventory’s (CTI) Validity (<1.5 *SD* below the mean) and Defensiveness (>1.5 *SD* above the mean) scales, as specified in the *CTI Manual* (Epstein, 2001). This left a total of 160 leader-follower dyads (*N* = 320).

Among the 160 dyads, the average age of leaders (*M* = 44.10, *SD* = 11.11) was higher than the average age of their followers (*M* = 36.52, *SD* = 12.47). Gender was similar in both the leader (male = 75, female = 85) and follower samples (male = 87, female = 72, missing = 1). Leaders had an average tenure of 5.75 years (*SD* = 6.37) in their current position. Followers had an average tenure of 3.19 years (*SD* = 3.73) in their current position. The participants were from a range of industries with the largest sub-groups being from retail, sales, and marketing (20.0%); healthcare (15.6%); engineering, mining, and construction (14.4%); and education (9.4%).

Links to the online questionnaire package were emailed to prospective participants. Leaders completed measures of CET information-processing styles designed and validated by Epstein and colleagues: the Rational-Experiential Multimodal Inventory (REIm) and CTI, and they also completed the Multifactor Leadership Questionnaire (MLQ-5X; self-report); followers completed the MLQ-5X (other report) and other measures that are not the focus of the present study (some of these results are reported by Curtis, 2018). Both leaders and followers also reported demographic information. Entry into a gift-voucher ($AU50) prize draw was offered as compensation for participation. Prize draw entry was recorded separately from the online questionnaire to maintain confidentiality.

#### Measures

##### The Rational-Experiential Multimodal Inventory

The REIm (Norris and Epstein, 2011) is a 42-item self-report questionnaire designed to measure preference for rational and experiential thinking. The *rational-thinking scale* contains 12 items (e.g., “I prefer complex to simple problems”). The *experiential-thinking scale* contains 30 items that represent three facets of experiential thinking: *imagination*, *emotionality*, and *intuition* (10 items per subscale). The imagination subscale measures respondents’ preference for visual stimuli and tendency to think visually (e.g., “I enjoy reading things that evoke visual images”). The emotionality subscale represents respondents’ emotional reactivity (e.g., “Everyday experiences often evoke strong experiences in me”). The intuition subscale represents respondents’ tendency to base their decisions on how they feel (e.g., “I often go by my instincts when deciding on a course of action”). An overall experiential thinking scale is calculated by combining these subscales. All items were presented in five-point Likert format with response options ranging from 1 (*Definitely False*) to 5 (*Definitely True*). The REIm’s scales had good Cronbach’s alpha internal consistency reliability coefficients except for emotionality (see Tables 1, 2). However, because the emotionality dimension is of theoretical interest, it was retained in analyses, but results for this scale are interpreted with caution.

**Table 1:** Descriptive statistics, Cronbach alpha internal consistencies, and correlations between leader-self-rated thinking styles and both follower-rated (FR) and leader-rated (LR) full-range leadership styles – Study 1.

				Transformational leadership (FR)	Transformational leadership (LR)	Transactional leadership (FR)	Transactional leadership (LR)	Passive-avoidant leadership (FR)	Passive-avoidant leadership (LR)
	*M*			2.84	3.08	2.42	2.47	0.82	0.79
		*SD*		0.74	0.45	0.68	0.53	0.66	0.45
			*α*	0.94	0.88	0.71	0.60	0.78	0.70
Rational thinking	3.81	0.51	0.86	0.06	0.36^**^	−0.02	0.27^**^	−0.05	−0.17^*^
Experiential thinking	3.40	0.37	0.80	0.20^*^	0.28^**^	0.17^*^	0.14	−0.10	0.10
Intuition	3.47	0.43	0.73	0.19^*^	0.18^*^	0.19^*^	0.09	−0.15	0.15
Emotionality	3.40	0.51	0.57	0.19^*^	0.22^*^	0.15	0.08	−0.18^*^	0.01
Imagination	3.33	0.54	0.71	0.07	0.21^*^	0.06	0.08	0.08	0.10
Global constructive thinking	3.61	0.44	0.90	0.13	0.43^**^	0.02	0.04	0.02	−0.37^**^
Emotional coping	3.45	0.58	0.92	0.01	0.22^**^	−0.07	−0.15	0.03	−0.29^**^
Behavioral coping	4.02	0.42	0.77	0.25^**^	0.60^**^	0.17^*^	0.34^**^	−0.14	−0.38^**^
Categorical thinking	2.57	0.57	0.79	0.04	−0.15	0.07	0.18^*^	0.09	0.18^*^
Esoteric thinking	2.19	0.71	0.85	0.02	−0.03	0.08	0.06	−0.08	0.22^**^
Naïve optimism	3.31	0.54	0.83	0.09	0.21^**^	0.11	0.25^**^	0.12	0.13
*n*				160	160	160	160	150	158

* *p* < 0.05;

** *p* < 0.01.

**Table 2:** Descriptive statistics, Cronbach alpha internal consistencies, and correlations between leader-self-rated thinking styles and both FR and LR full-range leadership styles – Study 2.

				Transformational leadership (FR)	Transformational leadership (LR)	Transactional leadership (FR)	Transactional leadership (LR)	Passive-avoidant leadership (FR)	Passive-avoidant leadership (LR)
	*M*			2.73	3.01	2.41	2.33	0.68	1.01
		*SD*		0.36	0.43	0.29	0.53	0.33	0.36
			α	0.93	0.86	0.71	0.65	0.81	0.62
Rational thinking	4.03	0.44	0.85	0.16	0.31^**^	0.06	0.02	−0.24^*^	−0.34^**^
Experiential thinking	3.15	0.37	0.81	0.03	0.17	0.06	0.24^*^	0.02	0.15
Intuition	3.23	0.51	0.79	−0.09	0.00	−0.07	0.23	0.07	0.29^*^
Emotionality	2.98	0.51	0.71	−0.06	0.11	0.09	0.12	−0.06	0.06
Imagination	3.24	0.56	0.75	0.20	0.25^*^	0.11	0.17	0.03	0.02
Global constructive thinking	3.61	0.36	0.82	0.13	0.15	−0.09	−0.18	−0.13	−0.18
Emotional coping	3.53	0.55	0.91	0.16	−0.02	−0.10	−0.34^**^	−0.17	−0.22
Behavioral coping	4.08	0.28	0.66	0.15	0.52^**^	0.08	0.19	−0.29^*^	−0.08
Categorical thinking	2.19	0.45	0.78	−0.06	−0.12	0.10	0.38^**^	−0.01	0.19
Esoteric thinking	2.08	0.47	0.73	−0.07	0.08	−0.04	0.23^*^	0.04	0.18
Naïve optimism	3.09	0.55	0.85	−0.02	0.12	0.04	0.34^**^	0.08	−0.35^**^

*n* = 74.

* *p* < 0.05;

** *p* < 0.01.

##### Constructive Thinking Inventory

The CTI (Epstein, 2001) is a 108-item, self-report measure used to operationalize aspects of constructive and automatic thinking. The CTI allows for the calculation of an overall global constructive thinking scale (29 items) and two constructive thinking factors: *emotional coping* (25 items; e.g., “I do not let little things bother me”) and *behavioral coping* (15 items; e.g., “I am the kind of person who takes action rather than just thinks or complains about a situation”). The emotional coping factor consists of four subscales: *self-acceptance*, *absence of negative overgeneralization*, *non-sensitivity*, and *absence of dwelling*. Behavioral coping consists of three subscales: *positive thinking*, *action orientation*, and *conscientiousness*. Facets of destructive thinking are represented on four scales: *personal superstitious thinking* (seven items; e.g., “If something good happens to me, I tend to assume it was luck”), *categorical thinking* (15 items; e.g., “I tend to classify people as either for me or against me”), *esoteric thinking* (13 items; “I believe in good and bad omens”), and *naïve optimism* (15 items; “I believe almost all people are basically good at heart”). The CTI also includes two lie scales: *defensiveness* (where people are presenting themselves excessively positively; eight items; e.g., “I am not bothered in the least when someone insults me for no good reason”) and *Validity* (a check of whether people are reading items carefully; eight items, e.g., “I never learned to read”). As noted, participants scoring outside the acceptable bounds for Validity or Defensiveness were excluded from the sample. Items were in a five-point Likert format with scores ranging from 1 (*Definitely False*) to 5 (*Definitely True*). The CTI scales had good Cronbach’s alpha internal consistentcy reliabilities (>0.70; see Table 2) except for personal superstitous thinking (0.60), which was thus ommitted from further analyses.

##### Multifactor Leadership Questionnaire

Both self-report and other-report versions of the MLQ-5X have 45 items and measure three leadership styles: transformational (20 items; e.g., “Instils pride in me for being associated with him/her”), transactional (eight items; e.g., “Provides me with assistance in exchange for my efforts”), and passive-avoidant (eight items; e.g., “Avoids getting involved when important issues arise”; Bass and Avolio, 1994, 1997). Items were responded to using a five-point scale anchored with 0 = “Not-at-all” to 4 = “Frequently, if not always.”

Transformational leadership has five subscales (*attributed-charisma*, *idealized-influence*, *inspirational-motivation*, *intellectual-stimulation*, and *individualized-consideration*). Transactional leadership has two subscales (*contingent-reward* and *management-by-exception-active*). Passive-avoidant leadership has two subscales (*management-by-exception-passive* and *laissez-faire leadership*; Avolio et al., 1999).

##### Demographics

Additional items were included in the questionnaires to assess biographical and contextual details of the sample: e.g., age, gender, industry, tenure, etc.

#### Planned Data Analysis

In both Studies 1 and 2, in order to test the hypotheses that thinking styles would be positively correlated with transformational leadership (H1: rational thinking; H2: behavioral coping, H3: imagination; and H4: emotionality), we calculated Pearson’s correlations between thinking styles and both self-rated and other-rated leadership styles. In addition, where multiple thinking styles correlated with transformational leadership, we regressed transformational leadership on thinking styles in order to examine the unique contribution of thinking styles to transformational leadership.

### Results

#### Data Screening

The data were screened to ensure that statistical assumptions for the analyses were met. The categorical thinking scale of the CTI was significantly positively skewed, and the skew was brought within normal bounds by a square-root transformation. Passive-avoidant leadership was significantly positively skewed for both the self-rated and follower-rated (FR) versions of the MLQ. For self-rated passive-avoidant leadership, the skew was corrected by removing three outliers, for follower-ratings, the skew was corrected *via* a logarithm transformation. No other statistical assumption breaches were found, and, for ease of interpretation, descriptive statistics are reported for the untransformed data. Principal components analysis with direct oblimin rotation was used to statistically investigate potential common methods bias for the self-report measures. There was little evidence for common methods bias, with the largest single component only accounting for 21.57% of the variance (Podsakoff et al., 2003). Descriptive statistics are reported in Table 1.

#### Correlational Analyses: Thinking Styles and Leadership Styles

Pearson’s product movement correlations were calculated between leaders’ self-rated and follower-rated leadership styles in order to assess the level of agreement in their ratings of leadership styles. Self-rated and follower-rated transformational leadership correlated significantly but weakly (*r* = 0.26, *p* = 0.001). However, self-rated and follower-rated transactional (*r* = 0.14, *p* = 0.089) and passive-avoidant leadership were not significantly correlated (*r* = 0.10, *p* = 0.23). Thus, there was a weak alignment between how followers evaluated their leaders’ styles and how leaders evaluated themselves.

Of more theoretical interest, correlations were calculated between the thinking-style scales of the REIm, CTI, and the leadership scales of the MLQ. Correlations between leadership styles and thinking styles are presented in Table 1. Partial support was found for H1, in that leaders’ preference for rational thinking was correlated with self-rated, but not follower-rated, transformational leadership. Consistent with H2, leaders’ preference for behavioral coping was positively correlated with both self-rated and follower-rated transformational leadership.

There were several weak, but significant, correlations between experiential thinking and its subscales and both self-rated and follower-rated transformational leadership. These results support H3 and H4, which expected positive correlations between transformational leadership and leaders’ preferences for imagination and emotionality.

In addition to the correlations predicted by the four hypotheses several weak, but significant, correlations were observed between some thinking style dimensions and self-rated and follower-rated transactional or passive-avoidant leadership, as shown in Table 1.

#### Regression Analyses

Because several thinking style variables correlated significantly with both self-rated and follower-rated transformational leadership, standard multiple regressions were calculated to assess the relative contributions of thinking styles to self-rated and follower-rated leadership separately. All thinking styles were entered into these regressions as predictors to allow for the potential detection of suppressed effects. To avoid a breach of the multicollinearity assumption, the global constructive thinking and total experiential thinking scales were omitted because they are constituted of items included in other scales. No breaches of the multicollinearity assumption were observed (all VIFs were <1.7). These analyses were supplemented with relative importance analysis, with relative weights calculated in order to determine the unique contributions of each thinking style variable as a predictor of leadership style (Tonidandel and LeBreton, 2011). The results of the regression analyses are presented in Table 3.

**Table 3:** Regression statistics and relative weights for transformational leadership regressed on significantly correlated thinking style variables.

Self-rated transformational leadership	*B*	*SE B*	*β*	Relative weight
Rational thinking	0.172	0.062	0.196^**^	0.08^*^
Intuition	0.114	0.075	0.110	0.02
Emotionality	0.198	0.063	0.227^**^	0.04^*^
Imagination	0.070	0.055	0.085	0.02
Emotional coping	0.056	0.054	0.072	0.03
Behavioral coping	0.510	0.079	0.482^**^	0.25^**^
Categorical thinking	−0.031	0.052	−0.039	0.01
Esoteric thinking	−0.071	0.043	−0.112	0.01
Naïve optimism	0.035	0.060	0.042	0.02
**Follower-rated transformational leadership**	***B***	***SE B***	***β***	**Relative weight**
Rational thinking	0.011	0.133	0.007	0.00
Intuition	0.246	0.163	0.143	0.02
Emotionality	0.180	0.136	0.125	0.02
Imagination	−0.018	0.120	−0.013	0.00
Emotional coping	−0.045	0.117	−0.035	0.00
Behavioral coping	0.476	0.171	0.272^**^	0.06^*^
Categorical thinking	0.027	0.111	0.021	0.00
Esoteric thinking	−0.047	0.093	−0.045	0.00
Naïve optimism	−0.011	0.129	−0.008	0.00

*n* = 160. Relative weight significance from 10,000 bootstrapped replications (see Tonidandel et al., 2009).

* *p* < 0.05;

** *p* < 0.01.

The regression analyses found that the thinking styles accounted for 48.2% of the variance in self-rated transformational leadership [*F*(9, 159) = 15.48, *p* < 0.001] and 11.8% of the variance in follower-rated transformational leadership [*F*(9, 159) = 2.24, *p* = 0.022]. Looking at the significance of the βs and relative weights in Table 3, although seven thinking style variables significantly correlated with self-rated transformational leadership, only three of these were significant predictors: rational thinking, behavioral coping, and emotionality. In addition, only behavioral coping was a significant predictor of follower-rated transformational leadership.

## Study 2

Study 1 found support for H2–H4 and partial support for H1, in that leaders’ preference for rational thinking was not significantly correlated with followers’ ratings of their transformational leadership. One methodological issue that may have influenced the outcomes of Study 1 is that leaders were rated by only one follower. For participant recruitment, leaders and followers had to agree to participate where the leader knew that a follower would be rating them confidentially. This recruitment method may have led to pairs being recruited who were happy to rate each other and, more generally, happy to work together. If this was the case, it may have led to overall more positive ratings of leaders by followers, thus restricting the range of leadership scores, which would potentially reduce the observed correlations between thinking styles and follower-rated leadership styles. In addition, as Study 1 is the first study that we are aware of to attempt to correlate leaders’ rational and experiential thinking with others’ ratings of their leadership, it is sensible to examine whether the results replicate. Because of these considerations, we surveyed thinking styles for a new set of leaders whose leadership was rated by multiple co-workers, including followers, peers, and supervisors. Study 2 tested the same hypotheses as Study 1.

### Method

#### Participants, Procedure, and Measures

The participants in Study 2 were recruited *via* Murdoch University Executive Education courses. Before participating in leadership development courses, 85 leaders completed the self-report REIm, CTI, and MLQ, and each leader was rated by between 3 and 12 co-workers (followers and/or peers and/or supervisors; *N* = 763) on the MLQ.

Participants were offered the choice to opt out of having their data anonymously used for research; five leaders opted out, their data were removed along with that of their 54 raters. Six further leaders were removed from the sample for the following reasons: one leader did not enter details that would allow their raters to be identified, one leader was rated by only one co-worker, two leaders’ scores fell outside the acceptable bounds of the CTI lie scale, and two fell outside the acceptable bounds of the CTI validity scale; these leaders’ 37 raters were also excluded. This left a total of 74 leaders. Thirty-one raters opted out of allowing their data to be used for research. Nine raters did not supply the name of the leader they were rating and 25 raters failed to complete the MLQ. This left a total of 607 raters with a minimum four raters per leader (*M* = 8.20 raters per leader).

The leaders were from three industry groups, emergency services (*n* = 50), environmental management (*n* = 20), and transport management (*n* = 4). The average age of leaders (*M* = 44.30, *SD* = 7.00) was slightly below the average age of their raters (*M* = 46.27, *SD* = 9.57). The sample of leaders (male = 60, female = 14) and raters were both predominantly male (male = 424, female = 126, missing = 57). Leaders had an average tenure of 3.84 years (*SD* = 4.84) in their current position. Raters had an average tenure of 3.75 years (*SD* = 5.06) in their current position.

The measures used in Study 2 were the same as in Study 1, with leaders completing the REIm, CTI, and MLQ and co-workers completing the MLQ about the leader. Murdoch University Executive Education was supplied by the Leaders’ organizations with a list of the leaders’ and their coworkers’ contract details. Murdoch University Executive Education staff then sent email invitations to these leaders and coworkers to complete the questionnaires.

### Results

#### Data Screening and Aggregation

To examine whether it was acceptable to aggregate the rater responses of leadership, we calculated ICC(1), ICC(2), and rwg for transformational, transactional, and passive-avoidant leadership. There was good agreement, based on the mean rwg values, for transformational (0.82), transactional (0.82), and passive-avoidant leadership (0.86; Bliese, 2000; Woehr et al., 2015). ICC(1) indicates that a significant amount of the raters’ responses in transformational leadership (22%, *p* < 0.01), transactional leadership (18%, *p* < 0.01), and passive-avoidant leadership (22%, *p* < 0.01) can be explained by raters’ rating the same leader. In addition, ICC(2) values were in line with values deemed acceptable for data aggregation; for transformational (0.70), transactional (0.64), and passive-avoidant leadership (0.70). Because of this, we calculated the average transformational, transactional, and passive-avoidant leadership score for each leader from their group of raters. The data were screened to ensure that statistical assumptions for the analyses were met, and no statistical assumption breaches were found. Principal components analysis with direct oblimin rotation was used to statistically investigate potential common methods bias for the self-report measures. There was little evidence for common methods bias, with the largest single component only accounting or 19.34% of the variance (Podsakoff et al., 2003). Descriptive statistics are reported in Table 2. As in Study 1, the personal superstitious thinking scale of the CTI was removed because of low internal consistency (*α* = 0.50), additionally, behavioral coping had an atypically low alpha, but it was retained in the analyses as a variable of key theoretical interest.

#### Correlational Analyses: Thinking Styles and Leadership Styles

Consistent with Study 1, self-rated and other-rated transformational leadership correlated significantly but weakly (*r* = 0.28, *p* = 0.014). However, self-rated and follower-rated transactional (*r* = −0.04, *p* = 0.762) and passive-avoidant leadership were not significantly correlated (*r* = −0.19, *p* = 0.104). Thus, there was a weak alignment between how followers evaluated their leaders’ styles and how leaders evaluated themselves.

Again, of more theoretical interest is the potential relationship between thinking styles and leadership styles. Pearson’s product movement correlations were calculated between leaders’ thinking styles and their self-rated and mean raters’ evaluations of their leadership styles, these results are presented in Table 2. H1–H3 gained partial support inasmuch as leaders’ preferences for rational thinking, imagination, and behavioral coping correlated positively with their self-rated transformational leadership. However, rational thinking and behavioral coping did not correlate significantly with other-rated transformational leadership. There was no support for H4 in that no significant relationships were observed between emotionality and transformational leadership, either self-rated or other-rated.

As in Study 1, several additional weak but significant correlations between thinking styles and transactional and passive-avoidant leadership were observed. Although no predictions were made concerning the relationship between thinking styles and these forms of leadership, some similarities emerged across the two studies.

#### Regression Analyses

As in Study 1, regression analyses were conducted on self-rated and other-rated transformational leadership to follow-up the correlational analysis, where all thinking styles were entered into these regressions as predictors to allow for the potential detection of suppressed effects. To avoid a breach of the multicollinearity assumption, the global constructive thinking and total experiential thinking scales were omitted because they are constituted of items included in other scales. No breaches of the multicollinearity assumption were observed (all VIFs were <1.65).

The regression analyses found that the thinking styles accounted for 30.2% of the variance in self-rated transformational leadership [*F*(7, 73) = 5.51, *p* < 0.001] but did not account for significant variance in other-rated transformational leadership [*F*(7, 73) = 1.01, *p* = 0.431]. For self-rated transformational leadership, only behavioral coping was a significant predictor in the regression (*β* = 0.50, *p* < 0.001), and no thinking style variables significantly predicted other-rated transformational leadership.

## Discussion

The results of the present studies make an interesting new contribution to research on the possible connection between individual differences in thinking styles and leadership styles as predicted by the CELM, and provide a better test of this model than previous studies. The present Studies partially supported H1, H2, and H3 that leaders’ preference for rational thinking, behavioral coping, and imaginative thinking would be positively correlated with transformational leadership. These relationships were observed in both Studies 1 and 2, but only when leadership style was self-rated. Moreover, of these three thinking style variables, only behavioral coping predicted self-rated transformational leadership in both studies when it was regressed on all of the thinking style variables.

H4 was partially supported, as leaders’ preference for emotionality in thinking was correlated with both self-rated and follower-rated transformational leadership in Study 1 but not in Study 2. In addition, emotionality was a significant predictor of self-rated transformational leadership in Study 1. A relationship between emotionality and transformational leadership was predicted by Cerni et al. (2014) in their CELM; however, this was untested when their model was proposed. Only three previous studies have examined this relationship, and these have found inconsistent evidence. Curtis et al. (2017) did not find a significant relationship between self-rated emotionality and self-rated transformational leadership in either of their studies. However, Curtis (2020) found that other-rated emotionality and other-rated transformational leadership correlated significantly, albeit weakly.

The use of other-raters of leadership, with leader-self-rated thinking styles, in the two studies reported in this paper is an important methodological advancement on most previous studies of leadership and CET-based thinking styles. Although the relationships between thinking styles and leadership styles support three of the four hypotheses when leadership styles were self-rated, support for these hypotheses was limited or absent when leadership style was rated by followers or co-workers. In Study 1, behavioral coping (H2), imagination (H3), and emotionality (H4) were correlated with follower-rated transformational leadership, and, of these, only behavioral coping significantly predicted follower-rated transformational leadership in the regression analysis. In Study 2, no thinking styles variables correlated significantly with other-rated transformational leadership.

The results of the present studies examining the relationship between transformational leadership, rational thinking, imagination, and behavioral coping are broadly consistent with those of previous studies. Four previous studies have found correlations between self-rated transformational leadership and leaders’ preferences for rational thinking (Cerni et al., 2008; Curtis et al., 2017). In addition, two previous studies have found correlations between self-rated transformational leadership and leaders’ preferences for imaginative thinking (Curtis et al., 2017). Moreover, in the one study, where followers rated both thinking styles and leadership styles, transformational leadership was positively correlated the rational thinking, imagination, and emotionality (Curtis, 2020). These previous studies, also found correlations between self-rated transformational leadership and behavioral coping, and two previous studies (Atwater and Yammarino, 1993; Dubinsky et al., 1995) have also found correlations between behavioral coping and other-rated transformational leadership. In sum, in the present studies, the correlation between leaders’ preference for rational thinking replicated only for self-rated transformational leadership but not for other-rated transformational leadership, while the correlation between leaders’ preference for behavioral coping and follower-rated transformational leadership was found in one of two studies.

### Behavioral Coping and Transformational Leadership: Theoretical and Practical Implications

As noted above, a relationship between behavioral coping and transformational leadership has been found in several previous studies (e.g., Dubinsky et al., 1995; Cerni et al., 2008; Curtis et al., 2017), and we found this in Study 1 when leadership was both self-rated and follower-rated and in Study 2 when leadership was self-rated. The replication of this relationship confirms that it is the most robust connection between thinking styles and transformational leadership of those predicted by the CELM.

Within the CELM, the expected theoretical relationship between behavioral coping and transformational leadership has been argued to be based on two mechanisms: behavioral coping increasing leaders’ adaptability and behavioral coping reducing leaders’ stress (Cerni et al., 2014). Similarly, Atwater and Yammarino (1993) argued that the focus on taking action to deal with problems would promote leader adaptability (Yukl and Mahsud, 2010). However, Atwater and Yammarino (1993) suggested another reason why behavioral coping may be related to effective leadership; namely, behavioral coping reflects emotional intelligence (Epstein, 1998).

The results of the present studies strengthen our confidence that behavioral coping and transformational leadership *are* related, however, the results do not add to our understanding of *why* they might be related from the various explanations offered in the literature. To disentangle the competing (or possibly complimentary) reasons for this relationship, several avenues of research are possible. First, if behavioral coping is related to transformational leadership because behavioral coping reduces emotional stress, a study such as those reported in this paper where stress is measured along with behavioral coping and transformational leadership could be undertaken. Such a study would allow researchers to examine whether stress mediates the relationship between behavioral coping and transformational leadership. By the same token, behavioral coping and emotional intelligence could be measured within the same study to determine whether behavioral coping’s relationship with transformational leadership is accounted for by emotional intelligence. In previous research, Reynolds and O’Dwyer (2008) found that coping generally predicted leadership effectiveness better than did emotional intelligence. However, their study did not assess transformational leadership specifically or separate behavioral coping from other forms of coping when assessing, it is impact on leader effectiveness against emotional intelligence.

Assessing the impact of behavioral coping on leader adaptability may require longitudinal research including the potential for interventions designed to increase behavioral coping. Some evidence exists that coaching leaders to reframe problems to use more effective coping, from the point of view of CET, increases their transformational leadership (Cerni et al., 2010a,b). Therefore, such interventions could be applied in research to examine whether coaching that promotes behavioral coping also promotes behavioral flexibility and adaptability, and subsequently transformational leadership. The potential for coaching to be effective in promoting behavioral coping not only offers a means by which the theoretical predictions of CELM can be tested, but also appears to be the most obvious practical implication of our findings.

Several studies have suggested that promoting reflective thinking about leadership, and leaders’ meta-cognition more generally, is related to improved leader performance and development (Cerni et al., 2010a; Steele and Day, 2018). Cerni (2015), in particular, has argued for the use of CET in guiding leaders’ reflection through coaching to better understand how their thinking is related to their behaviors as a leader. A key take-away message for leaders at all organizational levels is that reflection, with or without the aid of coaching, not just about what they do but about how they think, may be helpful making them better leaders.

### Rational Thinking and Transformational Leadership: Theoretical and Methodological Implications

It is important to consider the potential reasons for the absence of a relationship between leaders’ preference for rational thinking and others’ perceptions of them as transformational leaders, as this is a key theoretical prediction of the CELM (Cerni et al., 2014). There are three primary possibilities that may explain this result. First, it is possible that methodological short-comings of entirely self-report data in previous studies account for the relationship between rational thinking and transformational leadership – albeit that we replicated this relationship in our self-report data in both studies. Second, it is possible that the proposed theoretical connection between rational thinking and transformational leadership, which was not found in our studies when leadership was follower-rated, is incorrect. Third, it is possible that a relationship between leaders’ preference for rational thinking and transformational leadership exists, but was undetected in our studies. We discuss these possibilities below but remain agnostic regarding which explanation is superior.

As argued earlier in this paper, previous studies that have attempted to connect thinking styles and leadership styles have principally used self-report methods that may be subject to common methods bias. This was the key methodological rationale for the present studies’ recruitment of follower and coworker raters. Therefore, we must consider the possibility that the correlation between rational thinking and transformational leadership is only significant for the wholly self-rated data because of common methods bias and that the disappearance of this relationship for other-rated leadership may represent the elimination of this bias. However, there are several reasons to think that the relationship between rational thinking and self-rated transformational leadership is not exclusively an artifact of common methods bias. As per the suggestions of Conway and Lance (2010), this relationship has been found in studies using different measures and samples (i.e., both of the present studies, Cerni et al., 2008; Curtis et al., 2017), the variables in question are measured with good reliability and non-overlapping item content, and there is a plausible theoretical connection between the variables. In addition, as reported, principal components analysis suggested little evidence of a common methods factor in the current studies. Thus, although the potential influence of common methods bias in the correlations obtained for self-rated leadership and thinking styles is worth remembering, we are not convinced that it completely explains the lack of a correlation between rational thinking and other-rated transformational leadership.

The lack of expected relationships between thinking styles and other-rated leadership in Study 2 may be attributable to a combination of restriction of range and statistical power, rather than common methods bias. Looking at the descriptive statistics in Tables 1, 2, the variability of both follower-rated transformational leadership and leaders’ preference for behavioral coping were markedly smaller in Study 1 than in Study 2. This restriction of range may have reduced the study’s ability to detect correlations between the constructs.

As Akinci and Sadler-Smith (2013) reported, mean rational thinking was higher for leaders in higher-level leadership jobs. Thus, rational thinking may be associated with leadership, but the range of rational thinking may be constrained by leadership level. In our Study 2, in particular, leaders tended to hold similar level of appointment, which may account for the reduced variability of rational thinking scores. In addition, although the Study 2 leaders were rated by over 600 coworkers, the overall sample of leaders was under half that of Study 1. In the available literature, one study did detect significant correlations between self-rated leadership and thinking styles with a similar sized sample of leaders (Curtis et al., 2017). However, the increased error of measurement that is likely when comparing self and other ratings may have resulted in Study 2 being under-powered.

Theoretically, the CELM expects that rational thinking will be connected with transformational leadership because rational thinking contributes to adaptation by leaders, and the selection of a transformational leadership style is an effective adaptation (Cerni et al., 2014). The absence of a correlation between rational thinking and the global factor of transformational leadership does not support this theoretical connection proposed by the CELM. In addition, however, the CELM also suggests that rational thinking will be related to transformational leadership more directly, inasmuch as rational thinking is likely to align specifically with the transformational leadership factor of intellectual stimulation of followers (Cerni et al., 2014). The correlation between leaders’ rational thinking and follower-rated intellectual stimulation was not significant in Study 1 [*r*(160) = 0.06, *p* = 0.421] but was approaching significance in Study 2 [*r*(74) = 0.207, *p* = 0.077].

It is possible that a relationship exists between leader’s preference for rational thinking and the extent of their transformational leadership that other raters – especially followers – do not detect. Rational thinking, which engages a slower thinking system, may be associated with more deliberative and less action-focused decision-making that followers may perceive as evincing less transformational leadership. This possibility is in contrast to the relationship between leaders’ preference for behavioral coping and transformational leadership. Behavioral coping is a component of how constructively leaders’ experiential thinking is used (Epstein, 2001). Experiential thinking, in contrast to rational thinking, involves more frequent use of emotion and “going with one’s gut,” which may mean that leaders who prefer experiential thinking are more likely to engage followers’ emotions and make quicker decisions. Thus, behavioral coping may be evident in faster action-focused decisions than rational thinking. Furthermore, the relationship between leaders’ use of experiential information-processing and their leadership effectiveness is supported by a substantial body of research by Sadler-Smith and colleagues (e.g., Sadler-Smith, 2016).

## Limitations and Future Research

A limitation of the current studies was the low internal consistency reliabilities obtained for some measures that were of focal theoretical interest – emotionality (Study 1) and behavioral coping (Study 2). Norris and Epstein (2011) report satisfactory reliability the emotionality scale, which we found in Study 2, and all previous research that we can find using the CTI behavioral coping scale has reported alphas >0.7. However, the only other studies of which we are aware to use the emotionality scale in the context of leadership are studies of Curtis et al. (2017), and they too found low reliability for the emotionality scale. Curtis et al. (2017) suggest that a potential source of the low reliability is that the scale contains both negatively and positively valenced items – some asking about displays of exuberance and others sadness or anger. It is possible that leaders who display positive emotions are rated as transformational and those who display negative emotions are rated as not transformational, and, thus, a general construct of emotionality as operationalized in the REIm may be only weakly related to perceptions of transformational leadership. The emotionality scale of the REIm may require revision, and that splitting positive and negative emotionality may be desirable in assessing its connection with leadership (Curtis et al., 2017).

## Conclusion

The studies presented in this paper aimed to provide a more methodologically robust test of the CELM (Cerni et al., 2014) than has been published in the literature to date. Specifically, we assessed connections between thinking styles and leadership styles when leadership was both self-rated and follower-rated. Previous studies have not examined connections between leaders’ preference for rational and experiential thinking with their follower rated leadership styles. In contrast to the predictions of the CELM, we found that rational thinking did not correlate with follower-rated transformational leadership in either study, while experiential thinking in the form of emotionality and imagination correlated with both self-rated and follower rated transformational leadership in Study 1. Consistently with previous studies, we found significant relationships between leaders’ behavioral coping transformational leadership, including follower-rated transformational leadership in Study 1 but not in Study 2. The present studies’ results provide mixed support for the CELM’s proposed connections between individual differences in thinking styles and leadership styles. For researchers, the key message going forward is that more exploration is needed of the potential relationship between rational thinking and transformational leadership, which is theoretically predicted by the CELM but was not borne out in our studies’ results for follower-rated leadership. For practitioners, the takeaway message is that there was a relationship between leaders’ preference for behavioral coping and transformational leadership (whether self-rated or follower-rated), which has been replicated in several previous studies, across several contexts. As a semi-malleable trait, behavioral coping may be assessed for the purposes of selection and developed *via* coaching (e.g., Cerni et al., 2010a). Therefore, behavioral coping may prove a useful individual difference to continue to investigate in leadership recruitment and development.

## Data Availability Statement

The raw data supporting the conclusions of this article will be made available by the authors, without undue reservation.

## Ethics Statement

The studies involving human participants were reviewed and approved by Murdoch University Human Research Ethics Committee. Written informed consent for participation was obtained for this study in accordance with the national legislation and the institutional requirements.

## Author Contributions

GC conceptualized the studies, collected and analyzed the data, and wrote the manuscript. SW analyzed the multi-level data in Study 2, and reviewed and edited the manuscript. Both authors contributed to the article and approved the submitted version.

### Conflict of Interest

The authors declare that the research was conducted in the absence of any commercial or financial relationships that could be construed as a potential conflict of interest.
